# Engineering novel features for diabetes complication prediction using synthetic electronic health records

**DOI:** 10.3389/fgene.2025.1451290

**Published:** 2025-04-14

**Authors:** Daniel Voskergian, Burcu Bakir-Gungor, Malik Yousef

**Affiliations:** ^1^ Computer Engineering Department, Al-Quds University, Jerusalem, Palestine; ^2^ Department of Computer Engineering, Faculty of Engineering, Abdullah Gul University, Kayseri, Türkiye; ^3^ Department of Information Systems, Zefat Academic College, Zefat, Israel

**Keywords:** diabetes complications, synthetic electronic health records (EHRs), feature engineering, feature selection, predictive modeling, machine learning, risk prediction

## Abstract

Diabetes significantly affects millions of people worldwide, leading to substantial morbidity, disability, and mortality rates. Predicting diabetes-related complications from health records is crucial for early prevention and for the development of effective treatment plans. In order to predict four different complications of diabetes mellitus, i.e., retinopathy, chronic kidney disease, ischemic heart disease, and amputations, this study introduces a novel feature engineering approach. While developing the classification models, we utilize XGBoost feature selection method and various supervised machine learning algorithms, including Random Forest, XGBoost, LogitBoost, AdaBoost, and Decision Tree. These models were trained on synthetic electronic health records (EHR) generated by dual-adversarial autoencoders. These EHRs represent nearly 1 million synthetic patients derived from an authentic cohort of 979,308 individuals with diabetes. The variables considered in the models were the age range accompanied by chronic diseases that occur during patient visits starting from the onset of diabetes. Throughout the experiments, XGBoost and Random Forest demonstrated the best overall prediction performance. The final models, which are tailored to each complication and trained using our feature engineering approach, achieved an accuracy between 69% and 77% and an AUC between 77% and 84% using cross-validation, while the partitioned validation approach yielded an accuracy between 59% and 78% and an AUC between 66% and 85%. These findings imply that the performance of our method surpass the performance of the traditional Bag-of-Features approach, highlighting the effectiveness of our approach in enhancing model accuracy and robustness.

## Introduction

Diabetes mellitus, commonly known as diabetes, is a chronic metabolic disease characterized by insufficient insulin production by the pancreas (Type 1 diabetes) or ineffective utilization of the produced insulin by the body (Type 2 diabetes). Diabetes poses a critical global health concern, significantly affecting millions of people worldwide (Diabetes, 2024). It leads to substantial morbidity, disability, and mortality rates, especially in low- and middle-income countries (Diabetes, 2024). The global prevalence of diabetes surged from 108 million in 1980 to a staggering 537 million in 2021, and it is expected to increase to 783 million by the end of 2045 (Sun et al., 2022). According to the World Health Organization (WHO), diabetes and related kidney disease caused approximately 2 million deaths worldwide in 2019 (Diabetes, 2024).

Over time, increased glucose levels beyond the average level in the bloodstream affect various major human organs including the heart, eyes, blood vessels, nerves, and kidneys. It can result in serious, long-term, life-threatening health complications (Harding et al., 2019). The most prevalent types of complications in diabetes are classified into microvascular and macrovascular disorders. Microvascular disorders impact small blood vessels, including conditions such as nephropathy, neuropathy, and retinopathy. On the other hand, macrovascular disorders damage large blood vessels and encompass peripheral vascular disease, cerebrovascular disease, and ischemic heart disease (Cade, 2008). According to (Litwak et al., 2013), more than 50% of individuals with Type 2 diabetes are affected by microvascular complications, while more than 25% suffer from macrovascular complications. Diabetes complications account for over 68% of diabetes-related deaths (Sisodia and Sisodia, 2018).

Unfortunately, the early detection of diabetes-related complications poses a significant challenge, as symptoms often manifest in later stages (Deshpande et al., 2008). Therefore, for patients with diabetes, regular medical check-ups and routine screening such as dilated eye exams to detect eye disease, urine tests to check albumin levels for kidney functionality, and other tests are crucial. Alternatively, via analyzing routinely available patient-specific data (e.g., patient visit records tracing the diagnosis trajectories), scientists can assist clinicians in predicting patients at high risk of developing diabetes-related adverse outcomes. The development of such prediction models can alleviate the healthcare burden of diabetes, improve care pathways, enhance targeted and specialized preventive measures, consequently prevent or slow down the onset and progress of such diabetes-related complications (Girach et al., 2006; Mosa et al., 2022). Moreover, such systems can assist healthcare practitioners and doctors in terms of disease management and can help policymakers to save healthcare resources. Such efforts are essential for improving the overall quality of diabetes care (Alghamdi, 2023).

Nowadays, hospitals and healthcare providers widely adopt and deploy electronic health record (EHR) systems. In 2015, 84% of the hospitals in United States embraced EHR systems (Henry et al., 2016). Similar to paper records, EHRs store various forms of information about a patient during a hospital visit, including hospitalization details and patient-specific medical data, such as medical history, laboratory tests, vital signs, diagnoses, prescribed medications, administered interventions, and clinical outputs (Birkhead et al., 2015). Analyzing patient-specific data recorded in EHRs using data mining and machine learning algorithms can contribute to biomedical and clinical research enormously. It allows the analysis of the complex interplay between various extracted features from massive health record datasets. Moreover, it enables researchers to delve deeper into diseases, understand their progress, and uncover hidden patterns (e.g., risk factors), correlations, and decision rules from data. This transformative potential underscores the importance of EHR in advancing medical informatics and healthcare applications.

However, the wealth of EHRs are not always freely and easily accessible to the research community. The main reason is that EHRs often contain sensitive or regulated patient medical data, which impedes their optimal utilization (Keshta and Odeh, 2021). To prevent direct access to the real EHR data, healthcare organizations usually generate anonymized data using de-identification methods. These methods apply generalization and suppression operations to modify the patients’ attributes (e.g., k-anonymity, l-diversity, and t-closeness). However, these techniques are not robust against re-identification attacks and thus cannot entirely avoid private information disclosure (El Emam et al., 2015). Due to the complex legal, privacy, and security concerns surrounding medical data, the healthcare sector faces significant challenges in adopting information technology, data exchange, and interoperability. This urgency underscores the need for alternative methods.

A promising solution to the challenges of accessing real EHRs is the use of realistic synthetic data generated by deep generative models (Chen et al., 2021), notably Generative Adversarial Networks (GANs) (Goodfellow et al., 2014) and variational autoencoders (VAE) (Kingma and Welling, 2013). Synthetic data, being artificially created, do not have a direct correlation with real data (i.e., no synthetic record has a one-to-one relationship to the original patient’s records), making them resistant to re-identification (Lee et al., 2020). If synthetic data can accurately replicate the attributes of actual EHR data, it could significantly aid healthcare companies and researchers, eliminating the need for real data. The ability to predict relevant clinical endpoints from synthetic clinical health records opens the doors to enhanced decision-making, early introduction of personalized medical interventions, and improved patient outcomes, specifically in the context of diabetes care.

Along this line, here we propose a novel feature engineering approach to predict key diabetes-related outcomes such as retinopathy, chronic kidney disease, ischemic heart disease, and amputations. The models were trained using a large-scale dataset which includes nearly one million synthetic clinical health records. These records were generated using dual-adversarial autoencoders and they simulate realistic patient data by tracking the chronological sequence of diabetic patient visits starting from the onset of diabetes (Lee et al., 2020). The datasets include variables like age range and chronic conditions observed during patient visits and our generated models leverage diagnostic trajectories from these synthetic records to train and refine their predictive performance.

The contributions of this research effort can be summarized as follows:

- This study proposes a novel feature engineering approach for selecting representative features from raw synthetic EHR data.
- Using a synthetic EHR dataset, we evaluate the performance of various supervised machine learning models (i.e., Random Forest, XGBoost, LogitBoost, AdaBoost, and Decision Trees) to identify individuals (already diagnosed with diabetes) who are at increased risk of developing a complication. The generated models used a binary class variable to indicate whether a patient may develop one of four complications based on past diagnosis trajectories.
- We identify the dominant characteristics (features representing diagnosed chronic disease along with the age-range label) that may lead to diabetic complications through the application of feature selection methods such as XGB feature selection.

## Related work

Although several studies have been proposed in literature for predicting the onset of diabetes, predicting diabetes complications has received less attention. In this section, we review various studies that employed machine learning techniques to predict complications in diabetes patients using electronic health records (EHRs). Here the related studies are compared based on the datasets, machine learning models, studied complications, utilized features, preprocessing and imbalance handling techniques, performance metrics, and outcomes.

While we appreciate the value of identifying the “best” dataset, model, or approach, it is essential to note that such a comparison is inherently challenging and may not be entirely fair. Each study referenced in this section utilized different datasets with varying sizes, patient demographics, data collection periods, and feature sets, significantly impacting the model’s performance. Additionally, these studies’ objectives and clinical contexts differ, making a direct comparison less meaningful. Instead, our goal is to provide a comprehensive overview of how different studies have approached the prediction of diabetes complications. Given the diversity in methodologies and data, each study offers unique insights into different aspects of this complex problem.

### Datasets

Various studies have utilized diverse datasets to investigate complications associated with Type 2 Diabetes Mellitus (T2DM). For instance (Dagliati et al., 2018), utilized electronic health records of 943 T2DM patients collected over 10 years by Istituto Clinico Scientifico Maugeri (ICSM) in Italy. In contrast (Jian et al., 2021), relied on 884 records from the Rashid Center for Diabetes and Research (RCDR) in Ajman, UAE. Nicolucci et al. (2022) examined a much larger dataset of 147,664 patients, collected over 15 years from 23 Italian diabetes centers, while (Abaker and Saeed, 2021) focused on 644 EHRs from Alsukari Hospital. Mora et al. (2023) used a comprehensive administrative dataset from the Agency for Health Quality and Assessment of Catalonia (AQuAS), comprising 610,019 observations, and Mosa et al. (2022) leveraged Cerner’s “Health Facts EMR Data” with 148,109 unique patients from over 90 US healthcare systems. The largest dataset in this field was used in Ljubic et al. (2020), involving 1,910,674 patients from the Healthcare Cost and Utilization Project (HCUP) over 9 years.

### Machine learning models

Research studies have implemented a variety of machine-learning models to predict diabetes-related complications. Logistic regression (LR) was used in studies (Dagliati et al., 2018; Jian et al., 2021; Abaker and Saeed, 2021; Mora et al., 2023; Mosa et al., 2022). Support vector machines (SVMs) were employed in Dagliati et al. (2018), Jian et al. (2021), and Mosa et al. (2022). Random forest (RF) appeared in all studies except for Nicolucci et al. (2022). Decision tree (DT) models were utilized in Jian et al. (2021), Mora et al. (2023), and Mosa et al. (2022). Additionally, ensemble methods such as AdaBoost and XGBoost were applied in Jian et al. (2021), Nicolucci et al. (2022), Mora et al. (2023), and Mosa et al. (2022). Abaker and Saeed (2021) used k-nearest neighbor (k-NN), and Dagliati et al. (2018) included Naïve Bayes. Furthermore, Mosa et al. (2022) employed a multilayer perceptron. In contrast to these traditional machine learning models, the study presented in Ljubic et al. (2020) uniquely utilized deep learning models, specifically recurrent neural networks (RNN) with long short-term memory (LSTM) and gated recurrent units (GRU).

### Studied diabetes complications

The types of predicted diabetes complications varied across the research papers. Dagliati et al. (2018) investigated microvascular complications (nephropathy, neuropathy, and retinopathy) at different time intervals. In addition to these (Jian et al., 2021), included metabolic syndrome, dyslipidemia, diabetic foot, hypertension, and obesity. Nicolucci et al. (2022) examined eye complications, cardiovascular, cerebrovascular, peripheral vascular disease, nephropathy, and neuropathy. Mora et al. (2023) predicted nine diabetes-related complications, including hypertension, renal failure, myocardial infarction, cardiovascular, retinopathy, congestive heart failure, cerebrovascular, peripheral vascular, and stroke. Mosa et al. (2022) concentrated on eye diseases, kidney diseases, and neuropathy, while (Ljubic et al., 2020) explored ten complications, such as angina pectoris, atherosclerosis, ischemic chronic heart disease, depressive disorder, hearing loss, myocardial infarction, peripheral vascular disease, and the ones in (Dagliati et al., 2018).

### Utilized features

The numbers and types of features used in the above-mentioned studies varied significantly. Dagliati et al. (2018) included demographic data (age, gender, time to diagnosis), clinical data from EHRs (body mass index (BMI), glycated hemoglobin (HbA1c), hypertension, lipid profile, and smoking habit), and administrative data. Jian et al. (2021) analyzed 79 features, including medical tests, demographic attributes, and other-related variables (BMI, HbA1c, vitamin D, blood pressure, and diabetes types). Nicolucci et al. (2022) considered 46 features, focusing on demographic attributes, disease codes, laboratory tests, and prescription dates. Abaker and Saeed (2021) identified the best model using six out of 29 attributes (infection years, Blood sugar, swelling, diabetic ketoacidosis, speed of the heartbeat, and diabetic septic foot). Mora et al. (2023) included demographic information with diagnoses and procedures. Mosa et al. (2022) grouped 28,476 unique ICD9 and ICD10 diagnosis codes into clinically similar entities (broad categories), called CCS codes (285 unique groups) to train the machine learning models, while (Ljubic et al., 2020) used only ICD-9 codes (1023 codes were used after removing rarely or too frequently ones) and visit dates.

### Preprocessing and handling imbalance in datasets

Handling missing data and class imbalance were common preprocessing steps. Dagliati et al. (2018) used the missForest imputation algorithm and oversampling for minority classes. Jian et al. (2021) employed multiple imputation methods (MissForest, k-NN, and mean substitution) and handled imbalance using undersampling with k-means clustering and oversampling using the SMOTE algorithm. Nicolucci et al. (2022) used extra-values imputation and oversampling via SMOTE. Mosa et al. (2022) explored various balancing techniques, including oversampling, undersampling, and SMOTE. Papers Abaker and Saeed (2021) and Ljubic et al. (2020) focused on dimensionality reduction techniques. Abaker and Saeed (2021) employed sequential feature selection (SFS) to select significant features, while (Ljubic et al., 2020) applied singular value decomposition (SVD) to reduce the dimensionality of visits.

### Model performance evaluation

Comparing models is challenging since each study utilized different datasets with varying sizes and used different types and numbers of features. For the trained diabetic complications predictive models (Dagliati et al., 2018), reported an accuracy of up to 83.8%. Jian et al. (2021) achieved accuracy and F1-scores between 73.4% and 97.8%. Nicolucci et al. (2022) showed accuracy greater than 70% and AUC exceeding 80%. Abaker and Saeed (2021) reported logistic regression classifier to be the best performing model, with an accuracy of 81% and an F1 score of 75%. Mora et al. (2023) noted AUC values from 60% to 69% and accuracy rates between 60% and 75%. Mosa et al. (2022) indicated that SVM with oversampling was the most consistent, while (Ljubic et al., 2020) highlighted that the RNN GRU model achieved accuracy between 73% and 83%.

Although several models have been developed for predicting diabetes complications, the majority of these studies typically rely on a limited number of patient characteristics and are based on populations of limited size. This limitation is understandable due to the complex legal, privacy, and security concerns surrounding medical data usage, which restricts access. To address this problem, the presented study uses nearly one million synthetic electronic health records (EHRs) for diabetes patients generated using a Dual Adversarial AutoEncoder (DAAE). These synthetic EHRs allowed us to simulate a large-scale dataset that closely mirrors real-world diagnostic trajectories of diabetic patients, enabling the prediction of pertinent diabetes endpoints. In simpler terms, this synthetic approach offers a distinct advantage in terms of both data availability and variability, enabling us to overcome the limitations commonly faced by real-world datasets, such as restricted access or insufficient size. By leveraging this large and diverse dataset, our models are better positioned to generalize and provide valuable insights into diabetes complication prediction. Since the dataset used in this research had not been previously employed, and no prior performance metrics existed in the literature, this study establishes a baseline for future comparisons. Furthermore, while studies such as (Nicolucci et al., 2022; Mora et al., 2023; Mosa et al., 2022; Ljubic et al., 2020) used diagnostic codes as features, our study introduces a novel feature representation method that utilizes both diagnostic codes and age-range labels to train machine learning algorithms. The proposed approach enhances the predictive power and relevance of the models.

## Materials and methods

### Dataset

The dataset utilized in this study was sourced from the CAMDA 2023 Challenge (http://www.camda.info/). It includes an ordered sequence of pathologies for 999,936 synthetic patients. These synthetic records are based on an original cohort of 979,308 diabetes patients from the Health Population Database (Base Poblacional de Salud, BPS) in the Andalusian Health System, Spain. Generated using the Dual Adversarial AutoEncoder (DAAE) method (Lee et al., 2020), this synthetic dataset comprises highly realistic Electronic Health Records (EHRs). These EHRs precisely trace the diagnostic paths of diabetic patients, facilitating the prediction of critical diabetes-related outcomes based on past diagnosis trajectories.

The cohort of real patients was filtered by the CAMDA organizers and preprocessed according to the following criteria:

• Only patients diagnosed with diabetes from 2003 onwards were included.
• Patients lacking recorded dates of birth or age were excluded.
• Cases where diabetes and amputation were diagnosed in the same visit or in visits close to each other were removed.
• Sex was coded during the first visit, using “1111” for men and “2222” for women.
• Each visit includes an age-range label. The ranges are <10, 10–20, 20–30, 30–40, …, >90, with corresponding labels “1000,” “1010,” “1020,” “1030,” …, “1090.”
• Amputation, a low-frequency endpoint, was added and coded with the label “1999.”

The dataset for the 999,936 synthetic patients features an ordered list of visits per patient, with each visit including a list of co-occurring chronic diagnoses. Diagnoses were coded using the International Classification of Diseases (ICD-9) manual. The dataset comprises 83 distinct diagnostic codes and 10 age-range labels. The goal of this dataset is to identify strong relationships in diabetes-associated pathologies to enable the prediction of any pathology before it is diagnosed. Relevant endpoints for prediction include well-known pathological diabetes consequences such as: a) Retinopathy (encoded as “703”), b) Chronic kidney disease (encoded as “1401”), c) Ischemic heart disease (encoded as “910”), and d) Amputations (encoded as “1999”). In this study, we will refer to this dataset as sEHR Dataset.

### The proposed methodology

**FIGURE 1:**
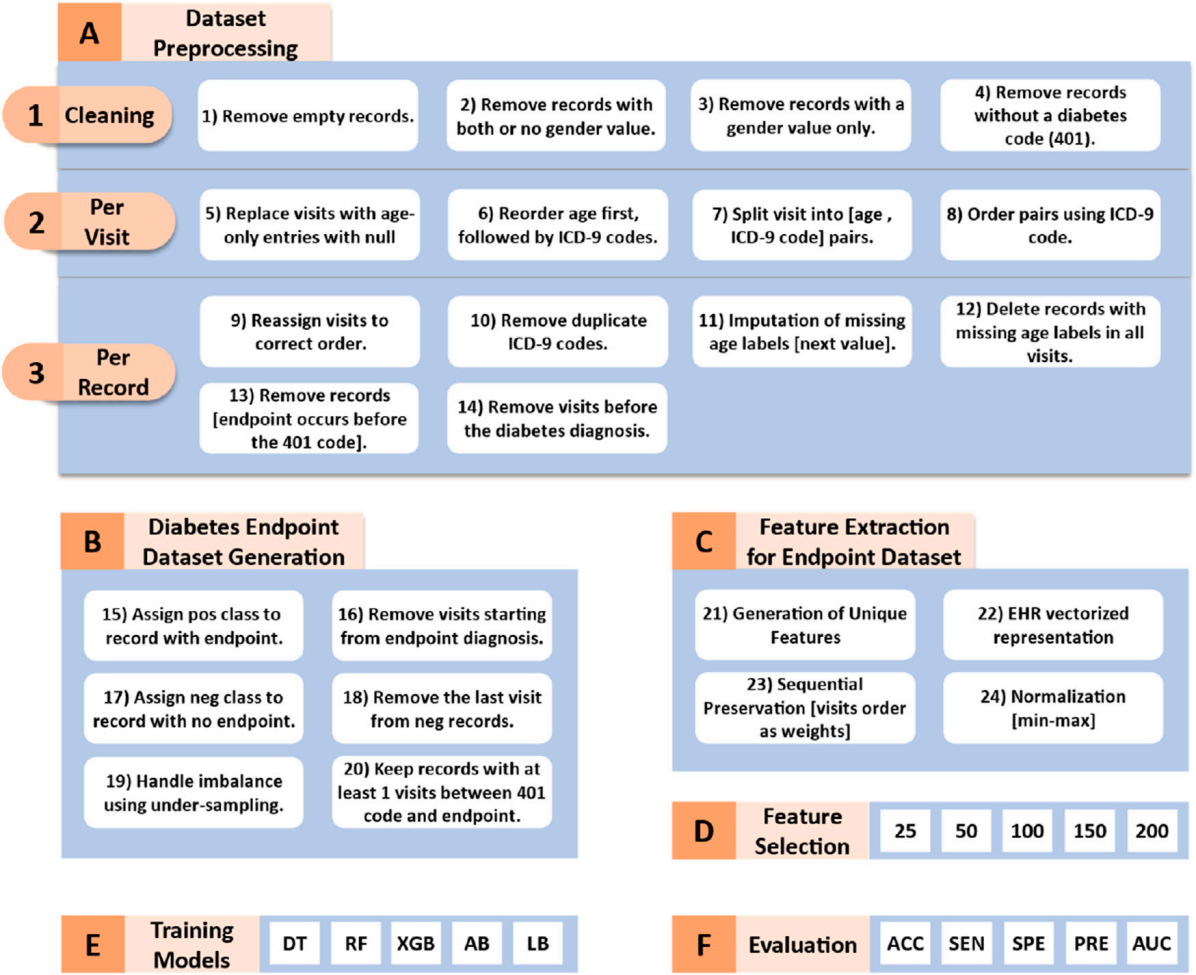
Workflow of the proposed methodology. DT (Decision Tree), RF (Random Forest), XGB (XGBoost), AB (AdaBoost), LD (LogitBoost), ACC (Accuracy), SEN (Sensitivity), SPE (Specificity), PRE (Precision), and AUC (Area Under the Curve).

Figure 1 illustrates the workflow of our proposed methodology, which can be divided into the following six key phases:

#### Dataset preprocessing

The original Synthetic Electronic Health Records (sEHR) dataset is structured in JSON format, where each record includes an organized list of visits for each patient. An exception is made for the first visit, which contains the gender label. Subsequent visits are accompanied by an age-range label, along with a corresponding list of chronic diseases (one or more pathology codes) that co-occur during that visit. The structure of a visit is represented as: Visit = [x, y] = [age-range label, pathologies codes]. This hierarchical arrangement provides a comprehensive overview of a patient’s medical history and facilitates the analysis of relationships between age groups, chronic diseases, and patient visits within the dataset. A snapshot of the raw data in the sEHR dataset, illustrating the data structure and accompanying descriptions, is provided in the Supplementary Material.

The sEHR dataset requires several preprocessing steps which are crucial for training the machine learning algorithms. These steps which are visualized in Figure 1A transform the dataset into a two-class format.

We start with converting the dataset from JSON format to a tabular format for easier manipulation and analysis. Next, as shown in Figures 1A–1, we performed a cleaning process which involved removing empty records, records with both or no gender value, records with gender value only (no registered visits), and records without diabetes code (401). All the patients included within this study are diagnosed with diabetes.

**FIGURE 2:**
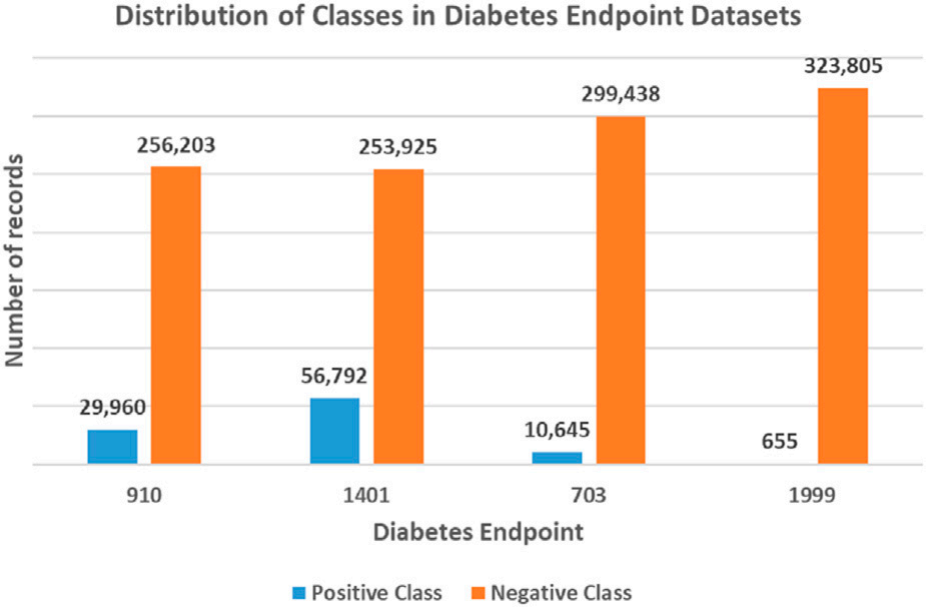
Unbalanced class distribution for each diabetes endpoint dataset. The four endpoints (diabetes complications) are encoded as following: 703: Retinopathy, 1401: Chronic kidney disease, 910: Ischemic heart disease, and 1999: Amputations.

Via iterating through each column, where each column represents one visit, we apply the following preprocessing tasks (as shown in Figures 1A–2). We replaced empty visits or visits that contained an age range label only with no pathology codes with null values. In some visits, the age range appears between or after the ICD-9 codes. We reordered them to ensure consistency among visits by placing the age-range label first. Afterwards, we split the original data structure, which included the age range label and multiple diagnostic codes, into individual pairs. Each pair consists of the age range label paired with one of the diagnostic codes. For example, [age range label, diagnostic code#1, diagnostic code#2] converted into [age range label, diagnostic code#1], [age range label, diagnostic code#2] pairs. Finally, we ordered the pairs within one visit in an ascending order according to the diagnostic codes.

**FIGURE 3:**
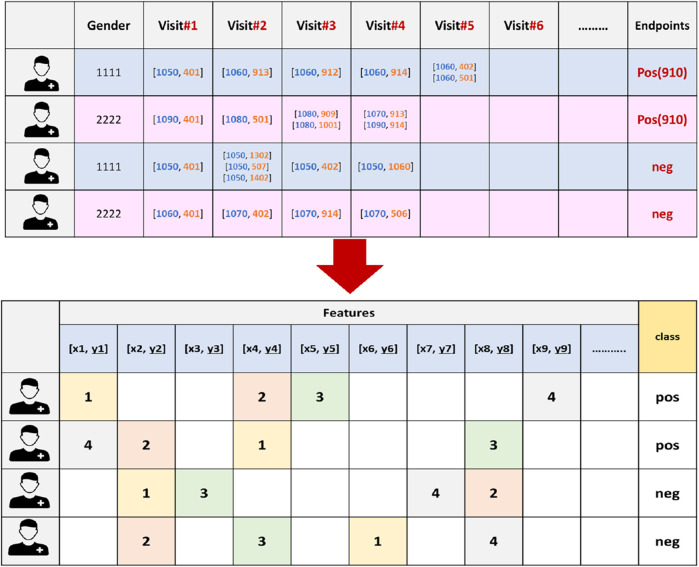
An example of the vectorization process for 910 endpoint dataset, where the features are extracted and the endpoints will serve as the class labels. X refers to an age-range label, while y represents a chronic disease code.

Subsequently, we iterated through each record, performing the following additional tasks (as shown in Figures 1A–3). *Firstly*, we eliminated null visits from the sequence and reassigned the subsequent visits to their correct order. For example, the sequence [visit#1, visit#2, null, visit#4, visit#5] was transformed into [visit#1, visit#2, visit#3, visit#4]. *Secondly*, only the first appearance of each diagnosis was kept (as they are chronic diseases). *Thirdly*, if a record contains a null age-range label in one of its visits, we handled missing values by selecting the next available age-range value to fill the gap. If the missing age range appeared at the last visit, we used the previous age range label. The entire record was deleted if all visits were missing the age label. *Fourthly*, we removed individual records from the analysis who were diagnosed with the required endpoints (developed a complication) before being diagnosed with diabetes. *Fifthly*, the visits that occurred before the diagnosis of diabetes were excluded. The fourth and fifth steps ensure that our analysis focuses on predicting the development of complications, specifically in patients with ongoing diabetes. By excluding data that precedes the diabetes diagnosis, we aim to train our models on information that is more relevant to the progression and management of diabetes-related complications, thereby improving the accuracy and effectiveness of our predictions.

After completing the sEHR dataset preprocessing, it was reduced from 17,745,138 to 324,575 records, with 896 entries representing patients’ hospital visits in the format of [age-range label, ICD-9 disease code]. At the end of these preprocessing steps (shown in Figure 1A) we ensure that the data is appropriately structured for the classification algorithms.

#### Generation of diabetes endpoint datasets

The sEHR dataset represents a multi-label classification task, where each EHR may be associated with multiple endpoints of interest (e.g., a patient may have multiple complications developed over time). Instead of treating this problem as a single multi-label problem, we decide to break it down into a set of binary classification tasks. A binary classification approach allows us to assess each complication individually, making it easier to explain the results and understand how specific features relate to the presence or absence of a single complication. This granular insight is more difficult to achieve via following a multi-label approach, where the interactions between labels could obscure the understanding of individual endpoint predictions. By employing binary classification approach, we can effectively address the complexity of multi-label classification and derive meaningful insights from the predictions obtained for each endpoint. Table 1 represents the distribution of diabetes endpoints in the sEHR Dataset. The numbers of records having single endpoints, two or more endpoints, three or more endpoints, and four endpoints are shown in Tables 1a–d, respectively.

**TABLE 1:** Distribution of Diabetes Endpoints in sEHR Dataset, showing occurrence of a) single, b) two or more, c) three or more, and d) four endpoints for a record. The four endpoints (diabetes complications) are encoded as following: 703: Retinopathy, 1401: Chronic kidney disease, 910: Ischemic heart disease, and 1999: Amputations.

a) Single endpoint	# of records
910	45,590
1401	46,475
703	15,701
1999	370

b) Two or More Endpoints		#of Records
910	1401	19,501
	703	4,871
	1999	187
1401	703	6,199
	1999	254
1999	703	91

c) Three or More Endpoints			# of Records
910	1401	703	1,676
	703	1999	29
	1401	1999	83
1401	703	1999	31

d) Four Endpoints				# of Records
910	1401	703	1999	11

To create a binary-class dataset for each endpoint, we extracted the required endpoint (diabetes-related complication) from each electronic health record to serve as the positive class label for the classification task. In order to avoid data leakage, if the patients were diagnosed with the complication under investigation during their first hospital visit, these records were excluded. Records that do not have the required endpoint are labeled as negative instances, with the last visit excluded to maintain a consistent prediction of a complication-free, no-endpoint scenario. Moreover, we retained records that included at least one hospital visit (temporal threshold) after the diagnosis of diabetes and before the occurrence of a diabetes-related complication, or the last visit for a patient without the specified complication.

To this end, the generated datasets in the current form result in a classification problem characterized by an unbalanced class distribution, with fewer positive class records than negative ones (refer to Figure 2). Specifically, the 910 endpoint cases accounted for 10.5%, the 1401 endpoint for 18.3%, the 703 endpoint for 3.4%, and the 1999 endpoint accounted for 0.2% of the patients in their respective datasets.

Leaving the datasets unaltered often results in poor predictive accuracy for the minority class, as the algorithm tends to predict the majority class more frequently (Japkowicz and Stephen, 2002). In order to address the issue of class imbalance, this study used a downsampling approach where the classes were balanced by randomly selecting the same number of negative class records as positive ones. Figure 1B summarizes the main tasks followed in this phase. At the end of this stage, we had four balanced datasets, one for each of the four complications. Table 2 provides the class distribution in each dataset after downsampling is applied.

**TABLE 2:** Class distribution of each diabetes endpoint in the binary class dataset after applying downsampling. The four endpoints (diabetes complications) are encoded as following: 703: Retinopathy, 1401: Chronic kidney disease, 910: Ischemic heart disease, and 1999: Amputations.

Endpoint	# of pos = # of neg	Total Records
910	29,960	59,920
1401	56,792	113,584
703	10,645	21,290
1999	655	1,310

Table 3 presents some statistics for each of the four diabetes complication records that exist in the generated binary class datasets. The last column contains the same measures for diabetes patients who do not have any of the studied complications.

**TABLE 3:** Statistics for each of the four diabetes complication records from the generated binary class datasets, including complication-free patient records. The four endpoints (diabetes complications) are encoded as following: 703: Retinopathy, 1401: Chronic kidney disease, 910: Ischemic heart disease, and 1999: Amputations.

	Label 910	Label 1401	Label 703	Label 1999	None
Number of Patients	29,960	56,792	10,645	655	188,941
Percentage (out of 324,575, the total number of preprocessed records)	9.2%	17.5%	3.3%	0.2%	58.2%
Number of visits per patient					
Max	10	10	10	9	11
Mean	2.87	3.20	2.72	3.92	2.74
Median	3	3	2	4	2
Std. deviation	1.10	1.29	1.03	1.70	1.01
Variance	1.22	1.65	1.06	2.91	1.02
Skewness	1.53	1.20	1.94	0.80	1.63
Kurtosis	2.75	1.50	5.38	0.18	3.14
ICD-9 codes					
Unique ICD-9 codes in the dataset	81	77	82	69	84
Unique features [age range, ICD-9 code] in the dataset	573	707	643	375	726
Max. Unique features [age range, ICD-9 code] per patient	20	17	22	17	26
Max. ICD-9 codes per patient					

Table 4 presents the top ten frequently observed features (the chronic diseases) categorized by age range. These features appeared most frequently among the records associated with the complication under investigation. These features were identified using the document frequency measure. The diabetes disease code (401) was excluded from the analysis because it is present in all records, given that the dataset is comprised of diabetes patients.

**TABLE 4:** Top ten frequently observed features (chronic diseases by age range) among the records associated with the complication under investigation. Features are color-coded based on chronic disease codes to highlight features belonging to the same disease category. The codes are defined as follows: 402: hyperlipidemia, 503: tobacco dependence, 507: Anxiety disorder, 604: Motor disorders without cerebrovascular accident (CVA), 906: extremity arteriopathy diseases, 910: Ischemic heart disease, 913: Hypertension, 914: Heart failure, 1,302: arthrosis/spondylosis. DF stands for document frequency for each feature. The four endpoints (diabetes complications) are encoded as following: 703: Retinopathy, 1401: Chronic kidney disease, 910: Ischemic heart disease, and 1999: Amputations.

910 Endpoint	
Feature	DF%
1060, 913	13.43
1070, 913	13.37
1060, 402	11.01
1060, 1302	10.09
1070, 1302	9.85
1070, 402	9.28
1050, 913	9.08
1070, 914	9.01
1050, 402	7.6
1060, 914	5.68

1401 Endpoint	
Feature	DF%
1070, 913	13.86
1060, 913	12.53
1070, 914	12.09
1070, 402	11.55
1070, 1302	11.45
1060, 402	11.16
1060, 1302	9.64
1080, 914	7.15
1070, 910	6.76
1060, 914	6.44

703 Endpoint	
Feature	DF%
1060, 913	12.91
1050, 913	12.01
1050, 402	10.1
1060, 402	9.98
1060, 1302	9.44
1070, 913	9.25
1050, 1302	7.4
1070, 1302	6.88
1070, 402	6.59
1050, 507	6.32

1999 Endpoint	
Feature	DF%
1060, 913	13.76
1050, 913	11.9
1060, 402	11.64
1050, 402	9.79
1060, 604	9.52
1060, 1302	8.73
1070, 913	8.73
1060, 906	8.2
1050, 503	7.94
1070, 1302	7.41

The analysis of top ten frequently appearing chronic diseases categorized by age range reveals significant patterns for each complication. Most chronic diseases appear when the individuals reach the age of fifty or older. Hypertension (913), hyperlipidemia (402), and arthrosis/spondylosis (1302) appeared among the top ten features for all four complications, indicating their prevalent and critical role in diabetes-related complications. Heart failure (914) was the most frequently observed feature for both Chronic kidney disease (1401) and Ischemic heart disease (910) complications. Ischemic heart disease (910) feature also appeared as one of the top 10 frequent features in Chronic kidney disease (1401) complication. Anxiety disorder (507) feature was significant for Retinopathy (703) complication. Motor disorders without cerebrovascular accident (CVA) (604), extremity arteriopathy diseases (906), and tobacco dependence (503) appeared in the top 10 frequent features list for Amputations complication (1999). These findings underscore the importance of these chronic conditions in the context of diabetes-related complications.

#### Feature extraction for each dataset

At this stage, we generated unique features (k) by identifying distinct combinations of age-range labels and pathology codes within the dataset. Gender (male, female) was also added to this list. Afterward, we converted each EHR into a (k + 2)-dimensional representation vector, capturing the comprehensive information pertaining to an individual patient. Within this representation, every dimension corresponds to a distinct [age range label, diagnostic code] recorded in the dataset.

Since the sequence in which these medical events occur is vital for understanding disease progression we carefully assigned each feature in the patient record a value that corresponds to the order of the hospital visits in which they occurred (refer to Figure 3). In our methodology, the order of the visits are utilized as weights. In other words, each feature in the patient’s record is not treated equally. Conversely, features from earlier visits were assigned lower weights, while those from later visits received higher weights, reflecting their temporal importance. This approach is critical for understanding how earlier and later visits contribute differently to predicting diabetes endpoints and enhances the model’s ability to learn patterns specific to diagnosis timing, which ultimately improves the accuracy and robustness of the predictions.

This transformation into a vectorized representation empowers efficient analysis and modeling of the EHR data, facilitating the discovery of meaningful patterns, relationships, and insights in the healthcare domain. Finally, we applied normalization to the final features to have a minimum value of 0 and a maximum of 1, using the max-min formula. This strategy helps to prevent higher values from disproportionately affecting the model’s learning process.

#### Feature selection

Following the feature extraction step, feature selection process is carried out using the XGBoost algorithm. In this context, selecting highly relevant features can minimize the computational power required to train an ML model and decrease computation time for the prediction process. Moreover, it can significantly enhance the model’s performance by removing irrelevant and noisy features. The top 25, 50, 100, 150, and 200 features were used to train various classification models. We decide to evaluate the generated models using reduced feature sets because our primary focus was on assessing the performance of the models with compact and highly discriminative feature subsets. Training on smaller feature sets provides insights into how efficiently our methodology selects and ranks the most relevant features for classification. Although we could extend the feature range, we opted to stop at 200 features for the following reasons:

• Performance Saturation: Preliminary experiments indicated that increasing the feature count beyond 200 did not significantly improve performance for most endpoints.
• Efficiency Considerations: Reducing the number of features aligns with our goal to develop a computationally efficient model that balances performance and resource usage.
• Focus on General Applicability: Most real-world applications benefit from models that perform well with fewer features, making this range more practical and interpretable for broader use cases.

#### Training phase

We start with decision trees (DT) classifier since they are interpretable and easy to understand. We incorporated more complex tree-based algorithms like Random Forest (RF), XGBoost (XGB), AdaBoost (AB), and LogitBoost (LB) to address potential overfitting problems. Although there is growing interest in deep learning models like RNNs and LSTMs as proposed in (Japkowicz and Stephen, 2002), in this study traditional machine learning algorithms are preferred due to the following reasons. *Firstly*, traditional models like RF and DT are highly interpretable. They provide insights into feature importance and decision paths, making predictions easily explainable to medical professionals and other stakeholders—a critical requirement in healthcare applications. *Secondly*, given the structured nature of our synthetic EHR dataset, tree-based and boosting algorithms are well-suited for tabular data, whereas deep learning models generally excel with unstructured data. *Thirdly*, traditional algorithms are computationally efficient, requiring fewer resources and less parameter tuning than deep learning models, while still delivering robust predictive performance. *Lastly*, tree-based models and boosting methods have consistently demonstrated high performance across various machine-learning tasks in the literature, further justifying their selection for this study.

#### Evaluation measures

To evaluate the performance of the constructed machine learning model, we employed two complementary evaluation approaches: Monte Carlo Cross-Validation (MCCV) and Partitioned Validation. In the MCCV approach, the dataset was randomly divided into 90% for training and 10% for testing. This splitting process was repeated K times, with performance being assessed by averaging all recorded metrics from the K tests.

In the Partitioned Validation approach, we utilized stratified random sampling to create five independent validation subsets, each representing 5% of the original dataset, totaling 25% that are reserved for validation. This method ensures consistent class distributions across subsets. The remaining 75% of the dataset was exclusively allocated for training purposes. For simplicity, we refer to this as “Partitioned Validation.” Table 5 provides details about the partitioning of different endpoint datasets for training and validation purposes.

**TABLE 5:** Partitioning the dataset for training and validation across endpoints.

Endpoint	# of Records in training Data (75%)	# of Records in validation data for each run (5%)	Total # of records in validation data (25%)	Overall dataset size (# of records)
910	44,940	2,996	14,980	59,920
703	15,967	1,064	5,320	21,287
1401	85,188	5,679	28,395	113,583
1999	982	65	325	1,307

The performance of the constructed models was evaluated using standard performance measures, including sensitivity, specificity, accuracy, precision, and area under the ROC curve (AUC).

## Experimental results

### Performance evaluation using cross -validation approach

For each diabetes-related complications, six primary experiments were conducted using the specified machine learning algorithms [DT, RF, XGB, AB, LB]. The initial experiment assessed the models using all available attributes in the dataset, while subsequent experiments utilized only the top 25, 50, 100, 150, and 200 attributes, respectively.

We employed repeated k-Monte Carlo cross-validation for model training (with k = 10) and conducted six repetitions for each feature subset, resulting in 60 experiments per ML model. With four complications and five machine learning algorithms, we realized 1,200 experiments (60 * 4 * 5) in total.

Table 6 summarizes the performance metrics of various machine learning models, trained to predict the four diabetes endpoints from patients’ visits using all features generated by our feature engineering approach on the diabetes-related endpoint datasets. The results presented in Table 6 were obtained using the Monte Carlo Cross-Validation (MCCV) approach.

**TABLE 6:** MCCV performance metrics of various machine learning models trained and tested using all features from the diabetes-related endpoint datasets. The four endpoints (diabetes complications) are encoded as following: 703: Retinopathy, 1401: Chronic kidney disease, 910: Ischemic heart disease, and 1999: Amputations.

# of Features	ML Model	Endpoint	Accuracy	Sensitivity	Specificity	F-measure	Precision	AUC
574	Adaboost	910	71%	**80%**	61%	73%	67%	80%
	DT		72%	71%	73%	72%	72%	72%
	LogitBoost		72%	78%	66%	73%	69%	81%
	RF		**75%**	69%	**81%**	73%	**78%**	83%
	XGBoost		74%	77%	70%	**75%**	72%	**84%**
644	Adaboost	703	72%	**80%**	65%	74%	69%	81%
	DT		72%	72%	71%	72%	72%	72%
	LogitBoost		74%	78%	70%	75%	72%	82%
	RF		**76%**	70%	**83%**	75%	**80%**	83%
	XGBoost		75%	78%	71%	**75%**	73%	**84%**
708	Adaboost	1401	67%	70%	64%	68%	66%	74%
	DT		66%	64%	69%	65%	67%	66%
	LogitBoost		67%	69%	66%	68%	67%	74%
	RF		**69%**	63%	**76%**	67%	**73%**	77%
	XGBoost		69%	**71%**	68%	**70%**	69%	**77%**
376	Adaboost	1999	68%	70%	67%	69%	68%	72%
	DT		63%	62%	65%	63%	64%	63%
	LogitBoost		69%	**71%**	67%	**70%**	69%	74%
	RF		67%	63%	70%	66%	68%	74%
	XGBoost		**70%**	69%	**71%**	70%	**71%**	**75%**

The highest performance metric values are highlighted in bold.

Among others, XGBoost and Random Forest emerged as the top-performing models, highlighting the effectiveness of tree-based ensemble algorithms for this type of problem. The final models, tailored to each complication, achieved an accuracy between 69 and 77 and AUC between 75 and 84. As noted in (Tan et al., 2023), an AUC of >0.75 signifies clearly useful discrimination performance. Our experimental findings underscore the value of leveraging ensemble approaches, combining multiple models to address individual model shortcomings. Both XGBoost and Random Forest aggregate the strengths of “weak” classifiers to construct robust final models. This approach significantly enhances overall performance by reducing variance and improving prediction accuracy.

### Performance evaluation using various feature subset sizes

**FIGURE 4:**
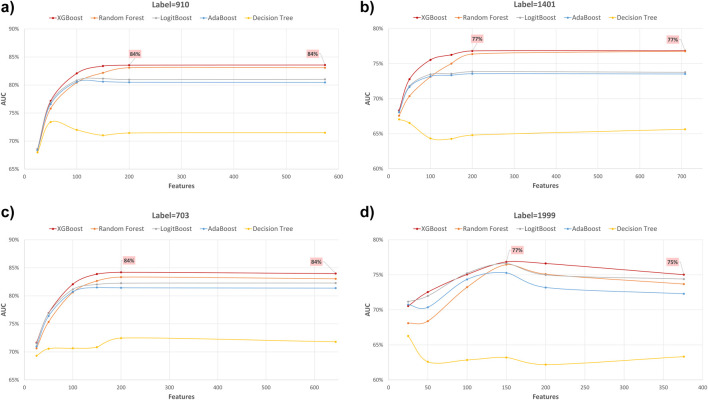
AUC performance metric of various machine learning models for predicting four diabetes endpoints using different feature subsets (extracted through XGB FS). The four endpoints (diabetes complications) are encoded as following: 703: Retinopathy, 1401: Chronic kidney disease, 910: Ischemic heart disease, and 1999: Amputations.

**FIGURE 5:**
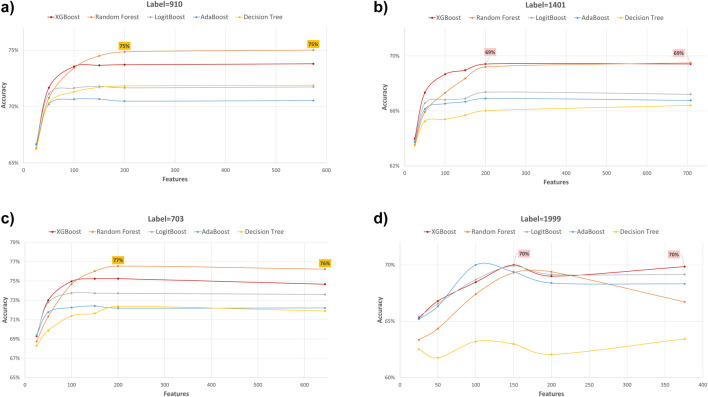
Accuracy performance metric of various machine learning models for predicting four diabetes endpoints using different feature subsets (extracted through XGB FS). The four endpoints (diabetes complications) are encoded as following: 703: Retinopathy, 1401: Chronic kidney disease, 910: Ischemic heart disease, and 1999: Amputations.

In our study, we employed XGB feature selection to examine the impact of reducing the number of features used during the training process (refer to Tables 7–11). We observed that most performance measures showed improvement as we increased the feature subset sizes in all models. As illustrated in Figures 4, 5, the models demonstrated a steady improvement in AUC and accuracy when utilizing feature subset sizes from 25 to 200 features. Beyond this point, both performance metrics remained relatively stable. However, for the Amputations (1999) endpoint, it peaked at 150 features and began to decrease after that. This phenomenon indicates that comparable or similar results can still be achieved by utilizing only a subset of attributes from the entire pool. This finding highlights the effectiveness of the FS in reducing the dataset dimensionality, simplifying the model, and reducing computational complexity.

**TABLE 7:** MCCV performance metrics of various machine learning models trained using 25 features extracted by XGB FS from the diabetes-related endpoint datasets.

ML Model	Endpoint	Accuracy	Sensitivity	Specificity	F-measure	Precision	AUC
Adaboost	910	67%	**86%**	47%	**72%**	62%	68%
DT		66%	86%	46%	72%	62%	68%
LogitBoost		**67%**	86%	**47%**	72%	**62%**	69%
RF		66%	86%	46%	72%	62%	68%
XGBoost		67%	86%	47%	72%	62%	**69%**
Adaboost	703	**69%**	84%	55%	**73%**	65%	71%
DT		68%	83%	53%	72%	64%	69%
LogitBoost		69%	83%	**56%**	73%	**65%**	72%
RF		69%	83%	54%	73%	65%	71%
XGBoost		69%	**84%**	54%	73%	65%	**72%**
Adaboost	1401	64%	**64%**	63%	**64%**	64%	68%
DT		63%	63%	64%	63%	64%	67%
LogitBoost		64%	63%	64%	64%	64%	68%
RF		64%	64%	63%	64%	64%	68%
XGBoost		**64%**	63%	**65%**	64%	**64%**	**68%**
Adaboost	1999	65%	70%	60%	67%	65%	71%
DT		63%	58%	**67%**	60%	65%	66%
LogitBoost		**65%**	65%	66%	65%	**67%**	**71%**
RF		63%	60%	66%	62%	65%	68%
XGBoost		65%	**72%**	59%	**67%**	65%	71%

The highest performance metric values are highlighted in bold.

**TABLE 8:** MCCV performance metrics of various machine learning models trained using 50 features extracted by XGB FS from the diabetes-related endpoint datasets.

ML Model	Endpoint	Accuracy	Sensitivity	Specificity	F-measure	Precision	AUC
Adaboost	910	70%	**80%**	61%	73%	67%	77%
DT		70%	73%	67%	71%	69%	73%
LogitBoost		71%	78%	65%	**73%**	69%	77%
RF		71%	74%	68%	72%	70%	76%
XGBoost		**72%**	73%	**71%**	72%	**71%**	**77%**
Adaboost	703	72%	**79%**	65%	74%	69%	76%
DT		70%	76%	63%	72%	68%	71%
LogitBoost		73%	76%	**70%**	74%	**71%**	77%
RF		71%	76%	66%	73%	69%	75%
XGBoost		**73%**	77%	69%	**74%**	71%	**77%**
Adaboost	1401	66%	**70%**	62%	**67%**	65%	72%
DT		65%	64%	66%	65%	65%	67%
LogitBoost		67%	69%	64%	67%	66%	72%
RF		66%	65%	67%	66%	66%	70%
XGBoost		**67%**	67%	**68%**	67%	**67%**	**73%**
Adaboost	1999	66%	64%	69%	66%	68%	70%
DT		62%	59%	65%	61%	63%	63%
LogitBoost		67%	62%	**72%**	65%	**69%**	72%
RF		64%	66%	63%	65%	64%	68%
XGBoost		**67%**	**66%**	68%	**67%**	67%	**73%**

The highest performance metric values are highlighted in bold.

**TABLE 9:** MCCV performance metrics of various machine learning models trained using 100 features extracted by XGB FS from the diabetes-related endpoint datasets.

ML Model	Endpoint	Accuracy	Sensitivity	Specificity	F-measure	Precision	AUC
Adaboost	910	71%	**81%**	61%	73%	67%	81%
DT		71%	71%	71%	71%	71%	72%
LogitBoost		72%	78%	65%	73%	69%	81%
RF		73%	71%	**76%**	73%	**75%**	80%
XGBoost		**74%**	77%	70%	**74%**	72%	**82%**
Adaboost	703	72%	**80%**	64%	74%	69%	81%
DT		71%	74%	69%	72%	70%	71%
LogitBoost		74%	78%	69%	75%	72%	81%
RF		75%	73%	**76%**	74%	**76%**	81%
XGBoost		**75%**	78%	72%	**76%**	74%	**82%**
Adaboost	1401	67%	**69%**	64%	67%	66%	73%
DT		65%	63%	68%	65%	66%	64%
LogitBoost		67%	68%	66%	67%	66%	73%
RF		67%	63%	**71%**	66%	**69%**	73%
XGBoost		**69%**	69%	68%	**69%**	69%	**76%**
Adaboost	1999	**70%**	**70%**	70%	**70%**	**70%**	74%
DT		63%	62%	65%	63%	64%	63%
LogitBoost		69%	68%	70%	68%	69%	**75%**
RF		67%	65%	**70%**	67%	69%	73%
XGBoost		68%	70%	67%	69%	68%	75%

The highest performance metric values are highlighted in bold.

**TABLE 10:** MCCV performance metrics of various machine learning models trained using 150 features extracted by XGB FS from the diabetes-related endpoint datasets.

ML Model	Endpoint	Accuracy	Sensitivity	Specificity	F-measure	Precision	AUC
Adaboost	910	71%	**80%**	61%	73%	67%	81%
DT		72%	71%	73%	71%	72%	71%
LogitBoost		72%	78%	65%	73%	69%	81%
RF		**75%**	70%	**79%**	73%	**77%**	82%
XGBoost		74%	77%	70%	**75%**	72%	**83%**
Adaboost	703	72%	**80%**	65%	74%	69%	82%
DT		72%	73%	70%	72%	71%	71%
LogitBoost		74%	78%	70%	75%	72%	82%
RF		**76%**	71%	**81%**	75%	**79%**	83%
XGBoost		75%	78%	72%	**76%**	74%	**84%**
Adaboost	1,401	67%	69%	64%	68%	66%	73%
DT		66%	63%	69%	65%	67%	64%
LogitBoost		67%	68%	65%	67%	66%	74%
RF		68%	63%	**74%**	66%	**71%**	75%
XGBoost		**69%**	**70%**	68%	**69%**	69%	**76%**
Adaboost	1999	69%	69%	70%	69%	70%	75%
DT		63%	63%	63%	63%	63%	63%
LogitBoost		70%	71%	69%	70%	70%	77%
RF		69%	68%	**71%**	69%	**71%**	76%
XGBoost		**70%**	**72%**	68%	**71%**	70%	**77%**

The highest performance metric values are highlighted in bold.

**TABLE 11:** MCCV performance metrics of various machine learning models trained using 200 features extracted by XGB FS from the diabetes-related endpoint datasets.

ML Model	Endpoint	Accuracy	Sensitivity	Specificity	F-measure	Precision	AUC
Adaboost	910	70%	**80%**	61%	73%	67%	80%
DT		72%	70%	73%	71%	72%	71%
LogitBoost		72%	78%	65%	73%	69%	81%
RF		**75%**	69%	**81%**	73%	**78%**	83%
XGBoost		74%	77%	70%	**75%**	72%	**84%**
Adaboost	703	72%	**80%**	65%	74%	69%	81%
DT		72%	73%	72%	73%	72%	72%
LogitBoost		74%	77%	70%	75%	72%	82%
RF		**77%**	70%	**83%**	75%	**80%**	83%
XGBoost		75%	78%	72%	**76%**	73%	**84%**
Adaboost	1,401	67%	70%	64%	68%	66%	74%
DT		66%	63%	69%	65%	67%	65%
LogitBoost		67%	69%	66%	68%	67%	74%
RF		69%	63%	**75%**	67%	**72%**	76%
XGBoost		**69%**	**71%**	68%	**70%**	69%	**77%**
Adaboost	1999	68%	67%	70%	68%	69%	73%
DT		62%	63%	61%	63%	62%	62%
LogitBoost		69%	70%	69%	**69%**	70%	75%
RF		**69%**	65%	**74%**	68%	**72%**	75%
XGBoost		69%	**70%**	68%	69%	69%	**77%**

The highest performance metric values are highlighted in bold.

The XGBOOST model consistently outperformed other models, achieving the highest AUC across all endpoints, even when using different feature subset sizes. This indicates that XGBOOST is particularly effective in distinguishing between positive and negative cases of diabetes complications. Conversely, the Decision Tree model consistently yielded the lowest AUC values across all endpoints and across different feature subset sizes, suggesting that it may not be the best choice for this prediction task.

On the other hand, the Random Forest model demonstrated high specificity and precision across the three endpoints (910, 1401, and 703) when employing feature subset sizes of 100 or more. In contrast, XGBOOST and LogitBoost achieved the highest values for these metrics with feature subset sizes less than 100, indicating that these models perform better with fewer selected features.

AdaBoost consistently achieved the highest sensitivity for the 910 and 703 endpoints across all feature subset sizes, indicating its effectiveness in correctly identifying positive cases of diabetes complications. For the 1401 endpoint, Adaboost achieved the highest sensitivity for smaller feature subset sizes (<=100), while XGBoost achieved the highest sensitivity for feature subset sizes greater than 100. For the 1999 endpoint, XGBOOST achieved the highest sensitivity for all feature subset sizes, demonstrating its robust performance across different endpoints.

Moreover, XGBOOST consistently attained the highest F1-score for the three endpoints (910, 1401, and 703) when the feature subset size was 100 or more, indicating its balanced performance in terms of precision and recall.

When comparing models constructed using 200 features for all endpoints, both XGBoost and Random Forest demonstrate superior performance. For 910, 1401, and 703 endpoints, XGBoost achieved the highest AUC and F1 score. On the other hand, Random Forest achieved the highest accuracy, specificity, and precision.

### Top predictors identified through XGBoost feature selection

Tables 12–15 present a summary of the top 25 high-impact predictors from each diabetes-related-complication dataset identified through XGBoost feature selection, ranked by their feature importance values as determined by each classification algorithm (Random Forest and XGBoost). These predictors are pairwise features, each comprising an age range label and a diagnostic code derived from patient visits within electronic health record (EHR) systems. These predictors can aid in identifying diagnosis codes and the corresponding age at occurrence, serving as risk factors for future diabetes-related diseases.

**TABLE 12:** Top 25 high-impact predictors for Ischemic heart disease (ICD9 = 910) complication of diabetes, identified by XGBoost feature selection and ranked by feature importance values from Random Forest and XGBoost.

RF	
Age Range	Diagnosed Disease
1080	Other organic mental disorder
1070	Other organic mental disorder
1060	Other organic mental disorder
1070	Arthrosis/spondylosis
1080	Acute cerebrovascular disease
1060	Anxiety disorder
1070	Acute cerebrovascular disease
1080	Chronic kidney disease
1070	Chronic kidney disease
1050	Arthrosis/spondylosis
1050	Anxiety disorder
1050	Other organic mental disorder
1080	Dementia
1070	Atrial fibrillation
1070	Anxiety disorder
1060	Chronic kidney disease
1070	COPD
1060	Acute cerebrovascular disease
1070	Dementia
1030	Diabetes
1060	Atrial fibrillation
1060	Motor disorders with no CVA
1080	Anxiety disorder
1060	Hypothyroidism
1020	Diabetes
XGBoost	
1030	Diabetes
1080	Other organic mental disorder
1020	Diabetes
1060	Other organic mental disorder
1070	Other organic mental disorder
1090	Other organic mental disorder
1050	Other organic mental disorder
1000	Diabetes
1060	Acute cerebrovascular disease
1050	Dementia
1050	Arthrosis/spondylosis
1010	Diabetes
1090	Dementia
1090	Chronic kidney disease
1050	Acute cerebrovascular disease
1050	Anxiety disorder
1080	Dementia
1060	Hypothyroidism
1090	Heart failure
1070	Acute cerebrovascular disease
1030	Anxiety disorder
1050	Hypothyroidism
1080	Chronic kidney disease
1080	Acute cerebrovascular disease
1060	Chronic kidney disease

**TABLE 13:** Top 25 high-impact predictors for Retinopathy (ICD9 = 703) complication of diabetes, identified by XGBoost feature selection and ranked by feature importance values from Random Forest and XGBoost.

RF	
Age Range	Diagnosed Disease
1070	Chronic kidney disease
1070	Atrial fibrillation
1060	Arthrosis/spondylosis
1080	Heart failure
1070	Other organic mental disorder
1070	Hyperlipidaemia
1080	Other organic mental disorder
1080	Chronic kidney disease
1070	Anxiety disorder
1080	Atrial fibrillation
1060	Anxiety disorder
1070	COPD
1060	Other organic mental disorder
1080	Hyperlipidaemia
1060	Chronic kidney disease
1060	Atrial fibrillation
1080	Ischemic heart disease
1070	Motor disorders with no CVA
1070	Extremity arteriopathy diseases
1070	Hypothyroidism
1080	Dementia
1070	Dementia
1060	Hypothyroidism
1080	Diabetes
1080	COPD
XGBoost	
1060	Other organic mental disorder
1080	Other organic mental disorder
1080	Diabetes
1070	Chronic kidney disease
1080	Dementia
1070	Atrial fibrillation
1070	Other organic mental disorder
1070	COPD
1070	Anxiety disorder
1090	Other organic mental disorder
1080	Chronic kidney disease
1080	Atrial fibrillation
1060	Anxiety disorder
1060	Hypothyroidism
1070	Dementia
1060	Chronic kidney disease
1080	COPD
1090	Heart failure
1080	Heart failure
1090	Chronic kidney disease
1050	Hypothyroidism
1090	Arthrosis/spondylosis
1060	Atrial fibrillation
1070	Hypothyroidism
1050	Other organic mental disorder

**TABLE 14:** Top 25 high-impact predictors for Amputation (ICD9 = 1999) complication of diabetes, identified by XGBoost feature selection and ranked by feature importance values from Random Forest and XGBoost.

RF	
Age Range	Diagnosed Disease
1060	Motor disorders with no CVA
1060	Arthrosis/spondylosis
1080	Diabetes
1070	Diabetes
1070	Atrial fibrillation
1060	Anxiety disorder
1060	Heart failure
1060	Atrial fibrillation
1070	Other organic mental disorder
1080	Heart failure
1070	Extremity arteriopathy diseases
1050	Motor disorders with no CVA
1080	Hyperlipidaemia
1060	Extremity arteriopathy diseases
1070	Tobacco dependence
1080	Acute cerebrovascular disease
1080	Arthrosis/spondylosis
1070	Alcohol dependence
1050	Extremity arteriopathy diseases
1050	Obesity
1050	Acute cerebrovascular disease
1040	Diabetes
1080	Dementia
1070	Mood disorder
1070	Hypothyroidism
XGBoost	
1080	Diabetes
1070	Diabetes
1080	Heart failure
1060	Atrial fibrillation
1050	Sequelae of cerebrovascular diseases
1080	Arthrosis/spondylosis
1060	Sequelae of cerebrovascular diseases
1050	Glaucoma
1050	Motor disorders with no CVA
1070	Prostate cancer
1040	Schizophrenia
1060	Arthrosis/spondylosis
1070	Other functional disorder
1070	Hypothyroidism
1060	Schizophrenia
1060	Other functional disorder
1070	Epilepsy
1050	Other functional disorder
1040	Diabetes
1060	Extrapyramidal disorder
1050	Extremity arteriopathy diseases
1060	Prostate cancer
1050	Extrapyramidal disorder
1040	Extremity arteriopathy diseases
1070	Atrial fibrillation

**TABLE 15:** Top 25 high-impact predictors for Chronic kidney disease (ICD9 = 1,401) complication of diabetes, identified by XGBoost feature selection and ranked by feature importance values from Random Forest and XGBoost.

RF	
Age Range	Diagnosed Disease
1040	Diabetes
1070	Anxiety disorder
1070	Hypertension
1060	Hypertension
1070	Other organic mental disorder
1080	Other organic mental disorder
1060	Arthrosis/spondylosis
1050	Arthrosis/spondylosis
1060	Anxiety disorder
1050	Anxiety disorder
1080	Atrial fibrillation
1070	Motor disorders with no CVA
1080	Acute cerebrovascular disease
1080	Anxiety disorder
1030	Diabetes
1070	COPD
1070	Dementia
1080	Dementia
1050	Diabetes
1070	Acute cerebrovascular disease
1060	Other organic mental disorder
1070	Diabetes
1060	Diabetes
1080	COPD
1060	Acute cerebrovascular disease
XGBoost	
1040	Diabetes
1030	Diabetes
1050	Diabetes
1080	Diabetes
1070	Diabetes
1060	Diabetes
1070	Other organic mental disorder
1090	Other organic mental disorder
1070	Anxiety disorder
1020	Diabetes
1080	Other organic mental disorder
1060	Anxiety disorder
1010	Diabetes
1060	Other organic mental disorder
1090	Arthrosis/spondylosis
1080	Dementia
1080	Atrial fibrillation
1000	Diabetes
1080	Anxiety disorder
1050	Anxiety disorder
1070	Hypertension
1050	Arthrosis/spondylosis
1060	Arthrosis/spondylosis
1090	Anxiety disorder
1090	Dementia

For comprehensive analysis, the top 50, 100, 150, and 200 high-impact predictors for each diabetes-related complication identified by the feature selection process are provided in the Supplementary Material, allowing further exploration of key factors influencing each endpoint.

### Comparative evaluation of feature engineering approaches

Finally, when comparing our feature engineering approach with the traditional Bag of Features (BOF), where each ICD-9 code, gender, and age-range label is considered as a separate feature, and their occurrences are used to build the record-feature matrix, significant differences emerge. Although the conventional BOF approach yields fewer distinct features—less than one hundred—, the comparative performance evaluation (summarized in Tables 16 **)** demonstrate the superiority of our approach. Our method not only performs better when training the model on all features but also excels when training on a subset size similar to the conventional approach. This improvement underscores the robustness of our feature engineering strategy in capturing more relevant and comprehensive information, leading to enhanced model performance.

**TABLE 16:** MCCV performance metrics obtained using XGBoost machine learning models combined with a) all features from the conventional approach (Bag of Features, BoF), b) all features as proposed in our approach, c) 100 features selected by XGB on our proposed diabetes-related endpoint datasets.

Endpoint	FS Model	# of Features	Accuracy	Sensitivity	Specificity	F-measure	Precision	AUC
910	BoF (all features)	94	70%	75%	64%	71%	68%	77%
	Proposed (all features)	574	**74%**	**77%**	**70%**	**75%**	**72%**	**84%**
	Proposed + XGB FS	100	74%	77%	70%	74%	72%	82%
1,401	BoF (all features)	94	66%	69%	64%	67%	66%	73%
	Proposed (all features)	708	**69%**	63%	**76%**	67%	**73%**	**77%**
	Proposed + XGB FS	100	69%	**69%**	68%	**69%**	69%	76%
703	BoF (all features)	94	73%	74%	71%	73%	72%	80%
	Proposed (all features)	644	75%	**78%**	71%	75%	73%	**84%**
	Proposed + XGB FS	100	**75%**	78%	**72%**	**76%**	**74%**	82%
1999	BoF (all features)	94	67%	66%	68%	67%	67%	73%
	Proposed (all features)	376	**70%**	69%	**71%**	**70%**	**71%**	75%
	Proposed + XGB FS	100	68%	**70%**	67%	69%	68%	**75%**

The highest performance metric values are highlighted in bold.

### Performance evaluation metrics across partitioned validation subsets

To provide a comprehensive assessment of the diabetes-related endpoint prediction models, we used the partitioned validation approach to calculate key performance metrics, including precision, specificity, sensitivity, accuracy, F1 score, and area under the curve (AUC), for each of the five independent validation subsets. Tables 17–20 showcase the performance metrics of Random Forest models trained using all features from the diabetes-related endpoint datasets and evaluated using the Partitioned Validation subsets.

**TABLE 17:** Performance metrics for endpoint 910 (ischemic heart disease) using random forest algorithm across partitioned validation subsets.

Validation Subset	Accuracy	Sensitivity	Specificity	F-measure	Precision	AUC
v1	76%	70%	82%	74%	79%	84%
v2	74%	70%	79%	73%	77%	83%
v3	74%	68%	79%	72%	76%	82%
v4	74%	68%	79%	72%	77%	82%
v5	74%	68%	81%	72%	78%	83%

**TABLE 18:** Performance metrics for endpoint 703 (retinopathy) using random forest algorithm across partitioned validation subsets.

Validation Subset	Accuracy	Sensitivity	Specificity	F-measure	Precision	AUC
v1	75%	69%	81%	74%	79%	83%
v2	77%	71%	82%	75%	80%	84%
v3	77%	72%	83%	76%	81%	85%
v4	78%	71%	85%	76%	83%	85%
v5	77%	70%	84%	75%	81%	84%

**TABLE 19:** Performance metrics for endpoint 1,401 (chronic kidney disease) using random forest algorithm across partitioned validation subsets.

Validation Subset	Accuracy	Sensitivity	Specificity	F-measure	Precision	AUC
v1	69%	62%	76%	67%	72%	76%
v2	68%	60%	76%	66%	72%	75%
v3	68%	60%	76%	65%	71%	74%
v4	69%	61%	77%	66%	72%	76%
v5	69%	61%	76%	66%	72%	75%

**TABLE 20:** Performance metrics for endpoint 1999 (amputations) using random forest algorithm across partitioned validation subsets.

Validation Subset	Accuracy	Sensitivity	Specificity	F-measure	Precision	AUC
v1	69%	72%	67%	70%	68%	76%
v2	72%	70%	75%	72%	74%	68%
v3	68%	63%	73%	66%	69%	76%
v4	59%	70%	51%	63%	59%	66%
v5	65%	70%	59%	67%	64%	69%

One can observe that across several performance metrics, there is a strong alignment between Cross-Validation results (as shown in Table 6) and Partitioned Validation results (as shown in Tables 17–20) particularly for endpoints having larger datasets (e.g., 910, 703, and 1401). The performance metrics obtained for endpoint 910 can be comparatively evaluated between two methods as follows:

• AUC: 83% (cross-validation) vs. 82%–84% (partitioned validation)
• F-measure: 73% vs. 72%–74%
• Precision: 78% vs. 76%–79%
• Specificity: 81% vs. 79%–82%
• Accuracy: 75% vs. 74%–76%

Here it worths to note that endpoint 910 has a large dataset. Similarly, for endpoint 703 the performance metrics remained consistent across the two evaluation methods:

• AUC: 83% (cross-validation) vs. 83%–85% (partitioned validation)
• F-measure: 75% vs. 74%–76%
• Precision: 80% vs. 79%–83%
• Specificity: 83% vs. 81%–85%
• Accuracy: 76% vs. 75%–78%

For endpoint 1401, we also observed a high degree of alignment:

• AUC: 77% (cross-validation) vs. 74%–76% (partitioned validation)
• F-measure: 67% vs. 65%–67%
• Precision: 73% vs. 71%–72%
• Specificity: 76% vs. 76%–77%
• Accuracy: 69% vs. 68%–69%

However, for endpoint 1999, which has a smaller dataset (including only 982 records), we observed greater variability in the stratified validation results. This variability is expected given the reduced sample size, which can introduce more fluctuations in model performance:

• F1 score ranged from 63% to 72%.
• AUC varied between 66% and 76%.

Despite this, the overall performance metrics for Endpoint 1999 were still in reasonable agreement with the cross-validation results (AUC: 74%). This suggests that, although the limited dataset introduces some variability, the model still demonstrates solid adaptability and consistent performance.

This comparative analysis highlights the robustness of our approach. The close alignment between Cross-Validation and Partitioned Validation results demonstrates the reliability of our models across evaluation strategies, indicating practical and generalizable performance.

## Conclusion

This study focused on developing prediction models for diabetes complications using a novel feature engineering approach, feature selection and various machine learning algorithms. Among others, XGBoost and Random Forest emerged as the top-performing models, showcasing the effectiveness of tree-based ensemble algorithms for this type of problem. The Random Forest and XGBoost models, customized for each complication, demonstrated an accuracy and F1-score ranging from 0.69 to 0.77, and an AUC between 0.75 and 0.85 using cross-validation, while the partitioned validation approach yielded an accuracy between 0.59 and 0.78 and an AUC between 0.66 and 0.85, indicating a clearly useful discrimination performance. This consistency between two evaluation methods not only underscores the reliability of our models but also instills confidence in the generalizability of our findings to real-world applications. Furthermore, it highlights that the performance of our models remains robust and is not overly reliant on the cross-validation approach alone.

Although we have achieved good results, external validation studies are necessary before considering their clinical implementation. This crucial step ensures that the models’ performance and generalizability are assessed in diverse populations or settings, confirming their reliability and effectiveness beyond the initial study cohort.

Since the dataset used in this research had not been previously employed, and no prior performance metrics existed in the literature, this study establishes a baseline for future comparisons. By conducting a comprehensive analysis of the dataset, this study aims to provide insights into its characteristics and potential challenges. Furthermore, this research sets a foundation for future studies to build upon and refine the models developed here.

For future work we aim to investigate the optimal number of hospitalizations occurring between the diagnosis of diabetes and the onset of each of the four complications for machine learning models to produce the best prediction accuracy. Additionally, we intend to employ longitudinal deep-learning models such as Long Short-Term Memory (LSTM) networks or multi-instance learning methods to model patient history. These approaches utilize time information that is mostly included in EHRs, which could lead to a more detailed and nuanced model.

## Data Availability

Publicly available datasets were analyzed in this study. This data can be found here: https://bipress.boku.ac.at/camda-play/the-camda-contest-challenges/#synthetic-health-records.
