# Correction: Engineering novel features for diabetes complication prediction using synthetic electronic health records

**DOI:** 10.3389/fgene.2025.1687832

**Published:** 2025-08-29

**Authors:** Daniel Voskergian, Burcu Bakir-Gungor, Malik Yousef

**Affiliations:** ^1^ Computer Engineering Department, Al-Quds University, Jerusalem, Palestine; ^2^ Department of Computer Engineering, Faculty of Engineering, Abdullah Gul University, Kayseri, Türkiye; ^3^ Department of Information Systems, Zefat Academic College, Zefat, Israel

**Keywords:** diabetes complications, synthetic electronic health records (EHRs), feature engineering, feature selection, predictive modeling, machine learning, risk prediction

Incorrect author assigned as Corresponding author

“Author Malik Yousef was erroneously assigned as corresponding author. The correct and sole corresponding author is Daniel Voskergian only.”

The original article has been updated.

