# Supplementary figures and images for: Engineering novel features for diabetes complication prediction using synthetic electronic health records
Source: Front Genet. 2025 Apr 14;16:1451290. doi: 10.3389/fgene.2025.1451290 (PMC12041673; doi:10.3389/fgene.2025.1451290)

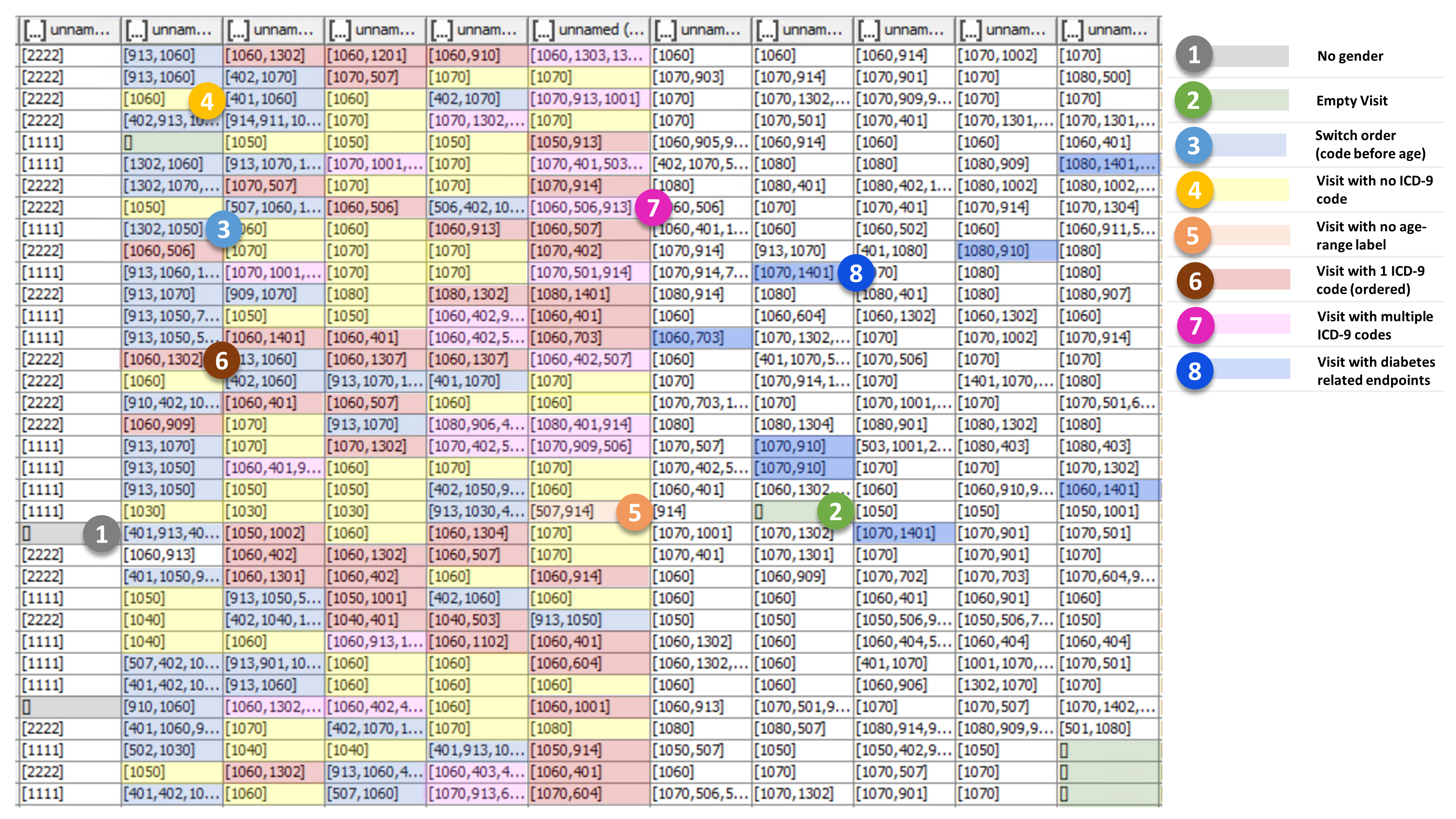

Supplement: Supplementary file 1 [file Image2.tif]

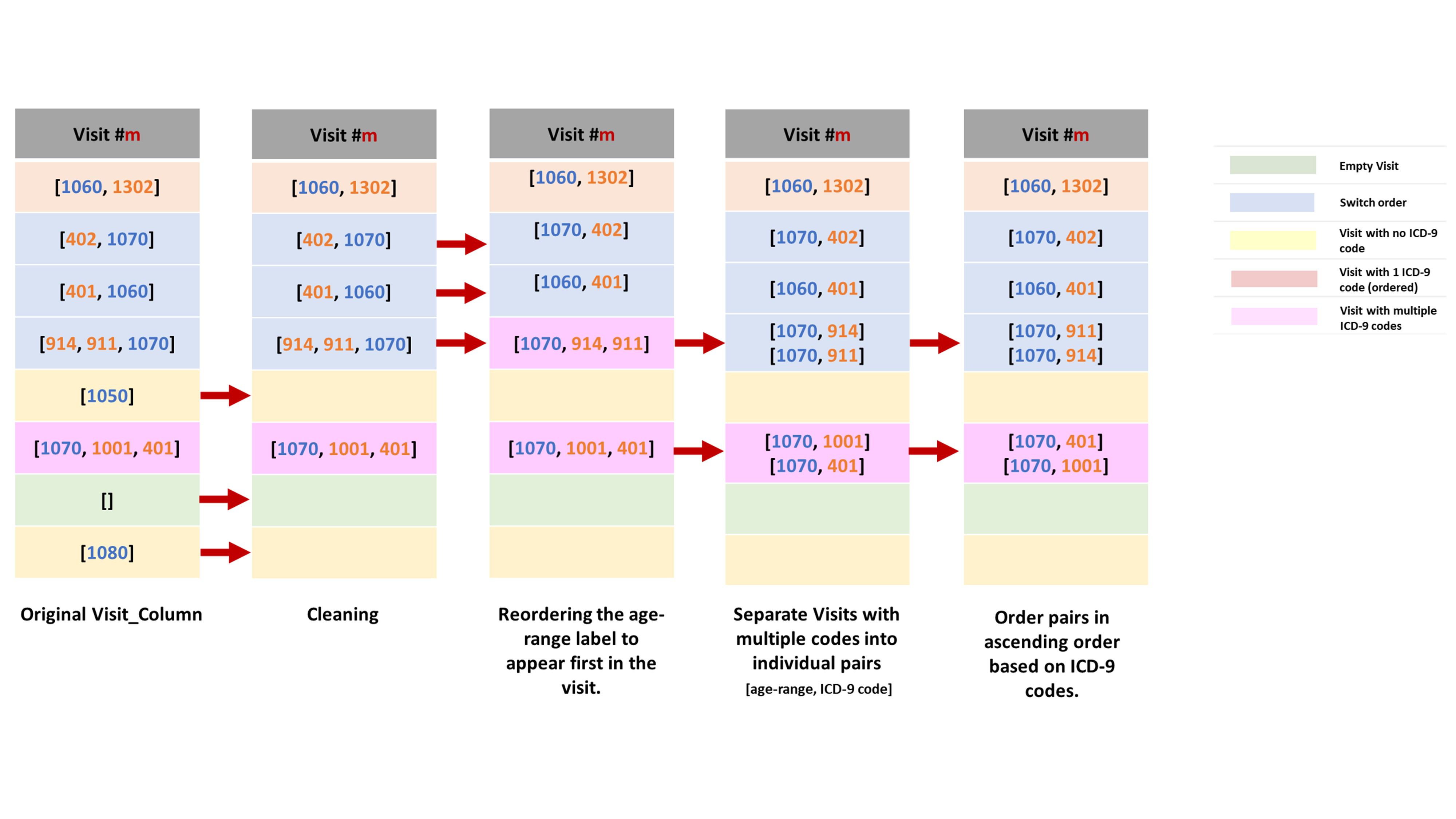

Supplement: Supplementary file 2 [file Image1.tif]
